# Evaluation of the Correspondence between the Concentration of Antimicrobials Entering Sewage Treatment Plant Influent and the Predicted Concentration of Antimicrobials Using Annual Sales, Shipping, and Prescriptions Data

**DOI:** 10.3390/antibiotics11040472

**Published:** 2022-04-01

**Authors:** Takashi Azuma, Takashi Nakano, Ryuji Koizumi, Nobuaki Matsunaga, Norio Ohmagari, Tetsuya Hayashi

**Affiliations:** 1 Department of Environment and Health Sciences, Faculty of Pharmacy, Osaka Medical and Pharmaceutical University, Takatsuki 569-1094, Japan; t.hayashi@soai.ac.jp; 2 Department of Microbiology and Infection Control, Faculty of Medicine, Osaka Medical and Pharmaceutical University, Takatsuki 569-8686, Japan; tnakano@ompu.ac.jp; 3 AMR Clinical Reference Center, National Center for Global Health and Medicine, Tokyo 162-8655, Japan; rykoizumi@hosp.ncgm.go.jp (R.K.); nomatsunaga@hosp.ncgm.go.jp (N.M.); nohmagari@hosp.ncgm.go.jp (N.O.); 4 Disease Control and Prevention Center, National Center for Global Health and Medicine, Tokyo 162-8655, Japan; 5 Department of Food and Nutrition Management Studies, Faculty of Human Development, Soai University, Osaka 559-0033, Japan

**Keywords:** antimicrobials, sewage influent, wastewater treatment plant (WWTP), predicted environmental concentration, national database of health insurance claims and specific health checkups of Japan (NDB)

## Abstract

The accuracy and correspondence between the measured concentrations from the survey and predicted concentrations on the basis of the three types of statistical antimicrobial use in Japan was evaluated. A monitoring survey of ten representative antimicrobials: ampicillin (APL), cefdinir (CDN), cefpodoxime proxetil (CPXP), ciprofloxacin (CFX), clarithromycin (CTM), doxycycline (DCL), levofloxacin (LFX), minocycline (MCL), tetracycline (TCL), and vancomycin (VMC), in the influent of sewage treatment plant (STP) located in urban areas of Japan, was conducted. Then, the measured values were verified in comparison with the predicted values estimated from the shipping volumes, sales volumes, and prescription volumes based on the National Database of Health Insurance Claims and Specific Health Checkups of Japan (NDB). The results indicate that the correspondence ratios between the predicted concentrations calculated on the basis of shipping and NDB volumes and the measured concentrations (predicted concentration/measured concentration) generally agreed for the detected concentration of antimicrobials in the STP influent. The correspondence ratio on the basis of shipping volume was, for CFX, 0.1; CTM, 2.9; LFX, 0.5; MCL, 1.9; and VMC, 1.7, and on the basis of NDB volume the measured concentration was CFX, 0.1; CTM, 3.7; DCL, 0.4; LFX, 0.7; MCL, 1.9; TCL, 0.6; and VMC, 1.6. To our knowledge, this is the first report to evaluate the accuracy of predicted concentrations based on sales, shipping, NDB statistics and measured concentrations for antimicrobials in the STP influent.

## 1. Introduction

The emergence and spread of antimicrobial-resistant bacteria (AMRB) is rapidly becoming an ongoing problem on a global scale [1,2,3]. The problem of AMRB has been assessed and addressed in medical facilities such as hospitals and livestock [4,5,6]. However, AMRB are widely detected in aquatic environments such as rivers, lakes, and seas [7,8,9], and sewage treatment plants (STPs), where various wastewaters are concentrated in sewage and have been noted as a potentially important source of AMRB [10,11,12].

Previous studies have detected antimicrobials, as well as AMRB, in wastewater [13,14]. These antimicrobials are mainly excreted from the body in the form of unchanged pairs that retain their original medicinal effects, and they are collected in STPs via the sewage system [15,16]. On the other hand, conventional wastewater treatment in STPs cannot completely remove chemical pollutants such as pharmaceuticals, and the treated water flows into the aquatic environments, such as rivers, lakes, and oceans [13,17]. The effects of these antimicrobial residues in these aquatic environments include not only toxic effects (growth inhibition, suppression of reproduction, and genotoxicity, etc.) on ecosystems but also on human health (allergy induction, dysbiosis on the gut flora, decreased immune system, and unexpected loss of healthy life expectancy, etc.) through drinking water sources, and these residues also potentially promote the development of new AMRB, thereby increasing the risk of human infection through exposure to microorganisms in the aquatic environments [8,18,19,20,21].

In today’s highly urbanized society, the sewerage system is highly developed [22]. Research has been conducted to find effective methods for predicting the concentration of AMRB in the STP influent, where wastewater is concentrated [23,24], and these methods have been supported by administrative agencies in Europe and the United States [25,26]. Previous research has evaluated a method for predicting the concentration of pharmaceuticals in the STP influent based on annual statistics of domestic production [26] and sales [25] of pharmaceuticals, such as prescription drugs (e.g., anti-inflammatory diclofenac, antihypertensive metoprolol, psychotropic carbamazepine, anticancer cyclophosphamide, and antimicrobial sulfamethoxazole), as well as over-the-counter drugs (OTCs). The order of magnitude has been mostly in agreement, although there are differences of approximately 10 times between the two types of predicted concentrations and the measured concentrations [27,28,29,30,31,32]. However, statistics on pharmaceutical use based on prescriptions and receipts on a nationwide scale are still in the process of being developed, even from a global perspective, and in many cases, there are no consolidated statistics, or they are not publicly published from the viewpoint of personal information; therefore, few studies exist, and much remains unknown [33]. Furthermore, previous studies have mainly investigated OTC drugs such as antipyretic analgesics, and further studies are essential for a comprehensive understanding of antimicrobials in the aquatic environments.

In Japan, two types of statistics on pharmaceutical use are available thus far. One type is statistics on annual sales volumes of pharmaceuticals [34], as provided by private businesses as part of market research, and the other type is statistics on annual shipping volumes of pharmaceuticals available on the market [35], as surveyed by the government. These two statistics are compiled as aggregate figures for all of Japan. In 2016, the Ministry of Health, Labour and Welfare released the National Database of Health Insurance Claims and Specific Health Checkups of Japan (NDB), which is a collection of information on medical receipts and specific health examinations by prefecture, excluding personal information [36]. Antimicrobials are exclusively used as prescription drugs, and their use as OTC drugs is prohibited in Japan. Therefore, it is possible to accurately grasp the actual use of antimicrobials, excluding those purchased without insurance coverage [37,38]. Under these situations, by applying the NDB database, it may be possible to predict the concentration of antimicrobials in the environment with a high degree of accuracy, taking into account the actual conditions of use. Nevertheless, to our knowledge, no study has yet predicted concentrations on the basis of the volumes of antimicrobials prescribed; moreover, the correspondence between predicted and measured concentrations is mostly unknown.

The aim of this study is, therefore, to evaluate the effectiveness of the estimation method for predicting antimicrobial concentrations in wastewater by assessing the estimation’s correspondence with the measured values obtained in the survey. This paper consists of the following structure. First, a seasonal, year-round monitoring survey was conducted for antimicrobials detected in influent from a STP located in urban areas of Japan. Next, the amount of antimicrobials used in the study area during the period studied was estimated using three statistics: domestic annual sales, shipping, and NDB statistics, and then the predicted concentration of antimicrobials in the STP influent from each amount used were calculated. Finally, the correspondence between the measured values obtained in the survey and the predicted values estimated from the amount used was verified, and the effectiveness of the method as a method for predicting the environmental impact associated with antimicrobial use was evaluated.

## 2. Materials and Methods

### 2.1. Microbes and Reagents

A total of 10 antimicrobials were surveyed on the basis of the previous report of concentration levels and frequencies of detection in hospital effluent, sewage, and river water reported both in Japan and around the world, and antimicrobial use in Japan as well [15,39,40]. The detailed names of the target antimicrobials are ampicillin (APL), cefdinir (CDN), cefpodoxime proxetil (CPXP), ciprofloxacin (CFX), clarithromycin (CTM), doxycycline (DCL), levofloxacin (LFX), minocycline (MCL), tetracycline (TCL), and vancomycin (VMC). The physicochemical properties and ratios of excretion as unchanged after ingestion are summarized in Table 1. All analytical standards were of high purity (>98%). Individual standard stock solutions at 10 mg/L were prepared in methanol and stored at −20 °C. All aqueous solutions were prepared with ultrapure Milli-Q water (18.2 MΩ·cm; Millipore Sigma, Watford, UK). Liquid chromatography–mass spectrometry (LC–MS)-grade methanol (>99%), formic acid (>99%), and acetone (>99%), analytical grade hydrochloric acid (>37%), ammonia (>30%), and ascorbic acid (>99%) were purchased from FUJIFILM Wako Pure Chemical Corporation (Osaka, Japan).

### 2.2. Sampling

STP influent was collected at a representative STP located in the Yodo River basin of Japan, which has a population of 17 million (14% of the Japanese population) [41,42]. At the time of this study, the STP disposed of municipal wastewater from 369,000 people, and the annual mean flow rate was 189,730 ± 5000 m^3^/day. The wastewater in the STP was treated with conventional activated sludge and then chlorine disinfection was applied. Sewage sludge produced in the treatment process is dewatered in a centrifuge and incinerated for disposal.

Sampling was conducted during 2019–2021: October 2019, September 2020, January 2021, and March 2021. This sampling frequency was on the basis of previous reports similar to the present research [31,32,43]. Identical manual sampling was adopted at a fixed sampling frequency because a composite sampler could not be used at the STP. Sampling was conducted on rain-free days, and no rainfall greater than 1 mm was observed during the two days before the sampling day [44].

A stainless-steel pail sampler was used to collect 100-mL samples of STP influent. Water samples were placed in sterilized glass bottle samples, and ascorbic acid (1 g/L) was immediately added as a preservative for antimicrobials [7,45]. All water samples were immediately transported to the laboratory in a cooler box (within 1 to 2 h), stored at 4 °C in darkness, and processed within 24 h.

**Table 1:** Properties of the target antimicrobials examined [46,47,48,49,50,51,52,53,54].

Antimicrobials	Molecular Formula	Molecular Mass (g/mol)	Structure	Excretion Rate (%)
Ampicillin (APL)	C_16_H_19_N_3_O_4_S	349.4	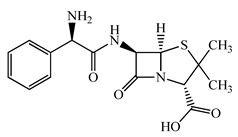	71 [46]
Cefdinir (CDN)	C_14_H_13_N_5_O_5_S_2_	395.4	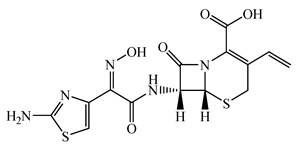	31 [47]
Cefpodoxime proxetil (CPXP)	C_21_H_27_N_5_O_9_S_2_	557.6	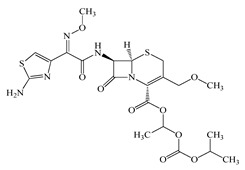	50 [48]
Ciprofloxacin (CFX)	C_17_H_18_FN_3_O_3_	331.3	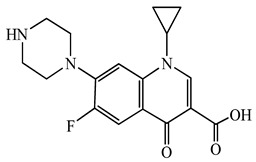	50 [49]
Clarithromycin (CTM)	C_38_H_69_NO_13_	748.0	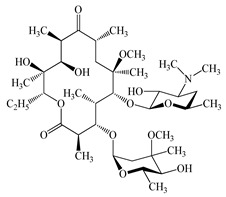	24 [50]
Doxycycline (DCL)	C_22_H_24_N_2_O_8_	444.4	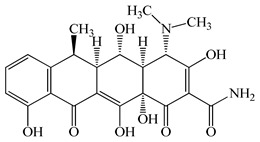	80 [51]
Levofloxacin (LFX)	C_18_H_20_FN_3_O_4_	361.4	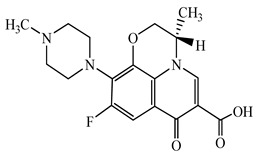	83 [52]
Minocycline (MCL)	C_23_H_27_N_3_O_7_	457.5	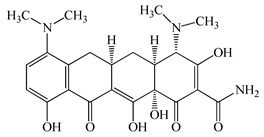	36 [51]
Tetracycline (TCL)	C_22_H_24_N_2_O_8_	444.4	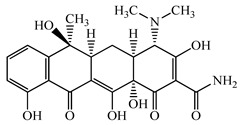	60 [53]
Vancomycin (VMC)	C_66_H_75_Cl_2_N_9_O_24_	1449.3	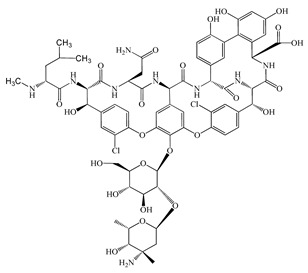	85 [54]

### 2.3. Analytical Procedures for Antimicrobials

The concentrations of target antimicrobials in the wastewater was determined by using a combination of solid phase extraction (SPE) and ultra-performance liquid chromatography–tandem mass spectrometry (UPLC–MS/MS), as described previously [7].

Briefly, 10 mL of STP influent was filtered through a glass-fibre filter (GF/B, 1-μm pore size, Whatman, Maidstone, UK). A known amount of each antimicrobial was spiked into two of the sample solutions for analysis to make a final concentration of 200 ng/L to account for matrix effects and loss during sample extraction [55]. The solutions were then subjected to two different preconditioned SPE cartridges (OASIS HLB, 200 mg; Waters Corp., Milford, MA, USA) at a flow rate of 1 mL/min; the cartridges had been preconditioned for each sample by being washed first with 3 mL of methanol and then with 3 mL of Milli-Q water adjusted to pH 3 with 1 N HCl. Similarly, the two unspiked sample solutions were removed and processed as above. All cartridges were washed with 6 mL of Milli-Q water preadjusted to pH 3 and then dried by a vacuum pump. Finally, the adsorbed antimicrobials were separately eluted with 3 mL of acetone and 3 mL methanol for one, and for the other with 2 mL of 10% (*v*/*v*) formic acid in acetone, 2 mL of 10% (*v*/*v*) formic acid in methanol, and 2 mL of 5% ammonia-methanol (*v*/*v*).

Each combined eluted solution was evaporated mildly to dryness under a gentle stream of N_2_ gas at 37 °C. The residue was solubilized in 200 μL of a 90:10 (*v*/*v*) mixture of 0.1% formic acid solution in methanol, and 10 μL of this solution was subjected for the UPLC system coupled to a tandem quadrupole mass spectrometer (TQD, Waters Corp.) equipped with an electrospray ionization source with positive and negative ion modes. Detailed information on the analytical parameters used for UPLC–MS/MS analysis is given in Table 2.

### 2.4. Method Validation

Six-point standard calibration curves were prepared for quantification ranging between 0.5 and 200 ng/mL. Individual linear calibration curves for each antimicrobial were obtained in the concentration range of 0.5 to 200 ng/mL (*r*^2^ > 0.99) by selecting the weighting factor of 1/x. Quantification was performed by subtracting the blank data from the corresponding data given by the spiked sample solutions to account for matrix effects and losses during sample extraction [56,57]. The recovery rates of antimicrobials in the STP influent ranged from 52% to 110% (Table 2). These profiles were generally similar to those reported in previous reports of pharmaceuticals in river water and wastewater samples [39,58,59]. The limits of detection (LODs) and limits of quantification (LOQs) were calculated as the concentrations at signal-to-noise ratios of 3 and 10, respectively, according to the methods applied to residual pharmaceuticals in environmental water samples [59,60]. These values are also summarized in Table 2.

### 2.5. Prediction of the Concentration of Antimicrobials in the Targeted STP Influent

The annual sales, shipping, and NDB statistical data are summarized in Table 3. Concentrations of antimicrobials in the targeted STP influent (*C*_(*STP*)*in*_) can be calculated by using annual sales volume summarized as an aggregate for all of Japan. (Equation (1)), as described below based on the previously reported estimation [31], assumes that antimicrobials are consumed equally every day [61,62] and that antimicrobial use is proportional to the population in each prefecture nationwide [25,27].

(1) $$C_{(STP)in}\;=\frac{D_{(Sales)total}\times E}{365}\times \frac{P_{STP}}{P_{Japan}}\times \frac{1}{W_{STP}}\times 10^{9}$$

where *D*_(*Sales*)*total*_ is the distribution of antimicrobials based on the annual shipping volumes in Japan considering pharmaceutical price and content (kg/year) (Table 3), *E* is the excretion rate of the target antimicrobials after administration as unchanged form (%) (Table 1), 365 is the number of days per year, *P_STP_* is the service population in the STP (number of persons) (369,000), *P_Japan_* is the population of Japan (126,227,000 persons) [63], *W_STP_* is the amount of influent water at the STP (189,730 m^3^/day) [64], and 10^9^ is a factor for unit conversion from kg/m^3^ to ng/L.

Concentrations of targeted antimicrobials in the targeted STP influent (*C*_(*STP*)*in*_) can be calculated by using annual shipping volumes summarized as an aggregate for all of Japan (kg/year) (Equation (2)) [31] and by assuming that all shipped antimicrobials are consumed equally every day [61,62] and that antimicrobial use is proportional to the population in each prefecture nationwide [25,26,27,28,30]:

(2) $$C_{(STP)in}\;=\frac{D_{(Ship)total}\times E}{365}\times \frac{P_{STP}}{P_{Japan}}\times \frac{1}{W_{STP}}\times 10^{9}$$

Furthermore, the concentrations of antimicrobials in the targeted STP influent (*C*_(*STP*)*in*_) can be calculated by using the annual NDB volumes summarized as prefecture-by-prefecture aggregate values (kg/year) (Equation (3)) and by assuming that all prescribed antimicrobials are consumed equally every day [37,61,62] and that antimicrobial use is proportional to the population in each prefecture nationwide [25,26,27,28,30]:

(3) $$C_{(STP)in}\;=\frac{D_{(Rceipt)prefecture}\times E}{365}\times \frac{P_{STP}}{P_{Pefecture}}\times \frac{1}{W_{STP}}\times 10^{9}$$

where *D*_(*Receipt*)_*_prefecture_* is the distribution of antimicrobials based on the annual NDB volumes in the prefectures (kg/year) (Table 3) and *P_Prefecture_* is the population of the prefecture (8,842,523 persons) [63].

## 3. Results and Discussion

### 3.1. Distribution of Concentrations of Antimicrobials in the STP Influent

**Figure 1:**
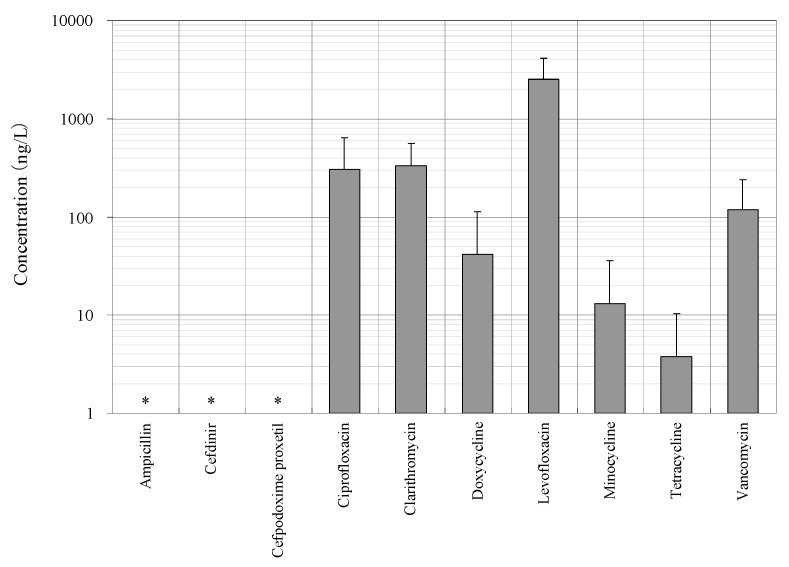
Measured concentrations of targeted antimicrobials in STP influents (*: Not detected).

Seven antimicrobials (CFX, CTM, DCL, LFX, MCL, TCL, and VMC) were detected at a wide range of concentrations from ng/L to μg/L levels (11 ng/L to a maximum of 4.3 μg/L) during the survey period in the STP influent (Figure 1). The detected concentrations of antimicrobials were similar to those previously reported [7,39,65]. The average detected concentrations of each antimicrobial were 309 ± 334 ng/L (CFX), 336 ± 223 ng/L (CTM), 42 ± 72 ng/L (DCL), 2536 ± 1617 ng/L (LFX), 13 ± 23 ng/L (MCL), 4 ± 7 ng/L (TCL), and 118 ± 120 ng/L (VMC). These results indicated that the antimicrobials used in the present study were used constantly throughout the year, with less seasonal fluctuation in use. This trend was mostly reasonable when trends in clinical use [61] were considered. In addition, pharmaceuticals detected in the environment, including antimicrobial components, are persistent in wastewater treatment processes and/or in the river environments [66,67] and remain in aquatic environments for long periods of time [68,69], the environmental risk of their inflow into the aquatic environments was considered to be a concern [70,71]. On the other hand, APL, CDN, and CPXP were not detected throughout the survey period. The reason for the lack of detection of these antimicrobials in the STP influent was related to the report that these antimicrobials are *β*-lactam antimicrobials with the common *β*-lactam structure in the structural formula and are easily degraded in the environment, being attenuated in a few hours to a few days [69,72], and were thought to be degraded in the sewage system before entering the STP.

### 3.2. Prediction of Annual Concentrations of Targeted Antimicrobials in the STP Influent

The estimated concentrations of each antimicrobial in the targeted STP influent on the basis of annual sales, shipping, and NDB volumes are shown in Table 4.

Concentrations calculated based on the shipping and NDB volumes were similar, and a significant difference (*p* < 0.05) was not observed in estimated concentrations between the shipping volumes and NDB volumes. In contrast, a significant difference (*p* < 0.05) was observed in estimated concentrations between the sales volumes and both shipping and NDB volumes; concentrations predicted on the basis of sales volumes were approximately two times lower than those predicted by using shipping and NDB volumes in the case of CDN and CPXP, seven times lower in the case of CTM, and approximately 20 times lower in the case of LFX and VMC. This difference probably occurred due to the statistics on shipping volumes and NDB volumes covering all pharmaceutical companies in Japan, but the statistics on sales volumes covered only those antimicrobials sold by major pharmaceutical companies, and targeted antimicrobials in this study are all sold by a large number of pharmaceutical companies as generic drugs [34]. These discrepancies in reporting are similar to those found in the previous studies [31,32].

### 3.3. Relationships between Measured and Predicted Concentrations of Antimicrobials

**Figure 2:**
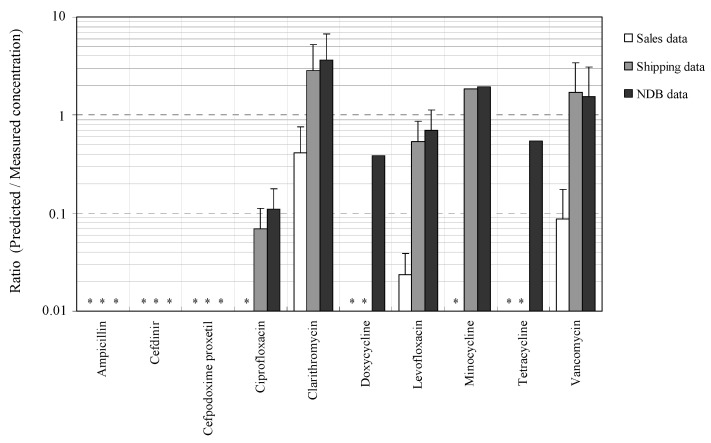
Correspondence ratios for predicted/measured concentration of target antimicrobials in STP influents (*: Not detected).

The correspondence ratios between the measured concentrations as described in Section 3.1 and the predicted concentrations estimated on the basis of sales, shipping, and NDB volumes as described in Section 3.2 as a ratio of predicted concentration/measured concentration are summarized in Figure 2.

The correspondence ratio between the predicted concentration calculated on the basis of sales volume and the measured concentration was 0.4 ± 0.4 for CTM, 0.02 ± 0.01 for LFX, and 0.1 ± 0.1 for VMC. The main reason for the differences in the predicted concentration and measured concentration based on the sales volume is probably the fact that CTM, LFX, and VMC are sold by many pharmaceutical companies as generic antimicrobials [34]. This trend supports the reports of a difference between NDB antimicrobial use and antimicrobial sales [38]. Interestingly, the orders of magnitude of the predicted and measured concentrations were generally in agreement for shipping and NDB volumes. The correspondence ratio between the predicted concentration calculated on the basis of shipping volume and the measured concentration was 0.1 ± 0.1 for CFX, 2.9 ± 2.4 for CTM, 0.5 ± 0.3 for LFX, 1.9 for MCL, and 1.7 ± 1.7 for VMC, and that on the basis of NDB volume and the measured concentration was 0.1 ± 0.1 for CFX, 3.7 ± 3.1 for CTM, 0.4 DCL, 0.7 ± 0.4 for LFX, 1.9 for MCL, 0.6 for TCL, and 1.6 ± 1.6 for VMC. The correspondence ratios generally agreed with the results in the present study, with previously reported results conducted in different regions based on the estimation from shipping volume (0.9 to 2.9 for CFX. 1.0 to 2.1 for CTM, and 18.3 for LFX [31,32]), although, in all cases, the effects of household disposal could not be excluded in the present estimations, as noted [73]. On the other hand, the fact that *β*-lactam antimicrobials APL, CDN, and CPXP were not detected in the effluent was considered to be due to the deviation from the predicted concentration due to their easy attenuation in the environment, as described in Section 3.1.

The measured concentration tended to be approximately 10 times higher than the predicted concentration in CFX in this study, even in the estimation from shipping and NDB volume. The low predicted concentration of CFX may originate from veterinary and livestock fields as well as from human use. Practically, the use of antimicrobials in animals is approximately twice as high as that in humans [74,75], and this trend is similar worldwide [76]. These results suggest that it would be important to attempt to evaluate veterinary antimicrobials with regional characteristics in addition to those used for human use to implement of risk assessment and management of the impact of antimicrobials in the aquatic environments.

### 3.4. Evaluation of Relationships between Concentrations of Antimicrobials Predicted by Using Sales, Shipping, and NDB Volumes

**Figure 3:**
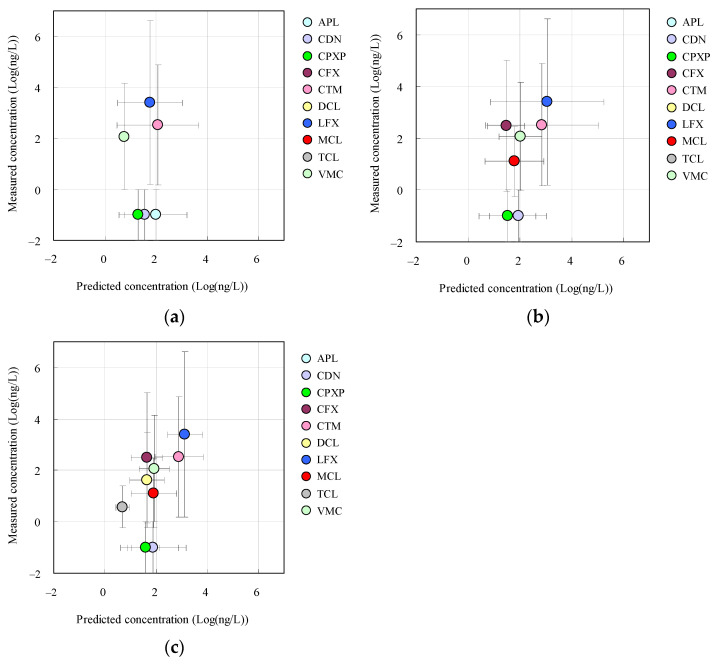
Relationship between correspondence ratios determined by using measured concentration values and the three types of predicted concentration values: (**a**) sales volume, (**b**) shipping volume, (**c**) NBD volume.

The correspondence ratios between the predicted concentrations based on the measured concentration and predicted concentrations on the basis of the sales, shipping, and NDB volumes are plotted against each other in Figure 3.

There was a positive correlation between the two (correlation coefficient *r* = 0.72 for shipping volume and 0.79 for NDB volume). It is therefore possible to calculate predicted concentrations with better precision by using statistical data on shipping volumes and/or NDB volumes, taking into account the actual conditions of use. On the other hand, in Japan, information on the shipping volumes of new pharmaceuticals used in the forefront of clinical practice is not available publicly for reasons associated with the patents applied to the manufacture and sales of these pharmaceuticals [35]. Therefore, the availability of statistical information on NDB volumes may be irreplaceable in some specific cases. For this reason, for the evaluation of new pharmaceuticals, improving the correspondence between measured and predicted values is an issue that needs to be addressed in the future.

The present method developed for predicting the concentrations of antimicrobials in the STP influent is simple, accurate, and precise. By taking into consideration the removal rates of pharmaceuticals by wastewater treatment processes at STPs and the dilution and attenuation rates during the river water flow where the influents are to be discharged, the use of the method used in this study should improve the accuracy of concentration prediction approaches targeting aquatic environments [77,78,79].

To the best of our knowledge, this is the first report to show the results of an evaluation of the accuracy of and correspondence between predicted concentrations based on sales, shipping, NDB statistics and measured concentrations for antimicrobials in the STP influent. These findings will contribute to a comprehensive understanding of the environmental risks associated with the presence of antimicrobials in the aquatic environments. In the future, the method developed here should be useful for the priority screening of antimicrobials and for conducting environmental risk assessments of antimicrobials that are yet to be studied in the aquatic environments. Furthermore, the introduction of advanced water treatment systems, such as ozonation [80,81], adsorption [82,83], membrane [84,85], electrochemical [86,87], and peracetic acid [88,89] are considered to be addressed in removing large amounts of pollutant loads including antimicrobial-resistant bacteria into the aquatic environments.

## 4. Limitations

The following limitations can be noted in this study. First, this study evaluated representative antimicrobials that are used in Japan and are considered to have higher concentrations detected in wastewater. Therefore, antimicrobials belonging to some medicinal classifications, such as sulfonamides [90] and aminoglycosides [91], were not investigated in this study, and for antimicrobials used for both human and animal use [92], there is a possibility that the discrepancy between the measured and predicted concentrations may be large, as mentioned in this report. Second, grab sampling was used for collecting wastewater at the STP in this study, and the concentration of antimicrobials in wastewater measured represents the concentration at a certain time of the day. Therefore, it would be possible that errors are due to diurnal variations caused by sampling methods compared to the continuous proportional sampling and flow proportional mode sampling [93,94].

Finally, the statistical aggregation of pharmaceutical use has limitations in that it does not include information on pharmaceuticals imported and purchased from abroad by individuals, as the regions where pharmaceuticals are prescribed may differ from the regions where they are actually used by patients, and it would also be affected by medication compliance, which assumes that all prescribed antimicrobials are taken [37,38]. Our results support the need for further, conclusive research by taking experimental, technical, regional customs, bias, and unknown factors into consideration.

## 5. Conclusions

The predicted concentrations of the representative antimicrobials in the STP influent of Japan by estimation from antimicrobial shipping and NDB volumes generally agreed with the measured concentrations with good accuracy. Additionally, although the concentrations predicted from the sales volumes were somewhat lower than those predicted from the shipping and NDB volumes, the orders of magnitude of the measured and predicted concentrations were roughly in agreement. These results indicate that the concentrations of antimicrobials in the STP influents can be estimated with high accuracy from two assets, antimicrobial shipping and NDB volumes. The fact that there is evidence that the use of antimicrobials has a direct impact on the environments is significant in terms of taking countermeasures for antimicrobials in the aquatic environments. Consideration of the removal rates during wastewater treatment processes at STPs and the dilution and attenuation rates in the receiving rivers should improve the accuracy of this method in predicting the concentrations of antimicrobials in river waters.

These findings highlight the effectiveness of the prediction of concentrations of antimicrobials in the STP influent. The importance of reducing the amount of antimicrobials before they are discharged into rivers was also evoked for maintaining both a clean environment and for human health. This study provides a new means of assessing the environmental risks associated with antimicrobials in aquatic environments.

## Figures and Tables

**Table 2:** LC–MS/MS parameters and validations of each antimicrobial.

Antimicrobials	Ionization Mode	Precursor Ion (*m*/*z*)	Product Ion (*m*/*z*)	Cone Voltage (V)	Collision Energy (eV)	Recovery (% (SD))	LOD (ng/L)	LOQ (ng/L)
Ampicillin (APL)	ESI+	350.2	*105.9*, 192.0	29	24	75 (33)	1.6	5.4
Cefdinir (CDN)	ESI+	369.2	170.0, *227.0*	30	20	72 (15)	0.2	0.8
Cefpodoxime proxetil (CPXP)	ESI+	557.5	*409.8*, 525.2	30	18	89 (5)	1.2	3.9
Ciprofloxacin (CFX)	ESI+	332.2	*288.2*, 314.2	40	25	57 (3)	0.7	2.2
Clarithromycin (CTM)	ESI+	748.2	316.6, *558.3*	38	18	74 (11)	0.5	1.6
Doxycycline (DCL)	ESI+	445.2	*428.3*	32	18	80 (9)	0.3	1.0
Levofloxacin (LFX)	ESI+	362.2	261.2, *318.2*	40	21	52 (6)	0.3	1.0
Minocycline (MCL)	ESI+	458.3	*441.0*	36	21	106 (18)	0.3	1.0
Tetracycline (TCL)	ESI+	445.2	*409.9*, 427.1	28	20	70 (20)	0.4	1.3
Vancomycin (VMC)	ESI+	724.2	82.9, *100.2*	17	18	110 (8)	0.6	1.9

Product ions in italics were used for quantification.

**Table 3:** Annual antimicrobial usage in the survey area [34,35,36].

Antimicrobials	Antimicrobial Usage (Mean (SD))		
	Sales * [34] (kg/year)	Shipping * [35] (kg/year)	NDB ** [36] (kg/year)
Ampicillin (APL)	3313 (562)	N.A.	182 (22)
Cefdinir (CDN)	2810 (181)	6707 (953)	410 (101)
Cefpodoxime proxetil (CPXP)	975 (276)	1588 (588)	130 (12)
Ciprofloxacin (CFX)	N.A.	1405 (241)	148 (13)
Clarithromycin (CTM)	11,592 (3970)	71,850 (15,086)	5499 (63)
Doxycycline (DCL)	N.A.	N.A.	93 (9)
Levofloxacin (LFX)	1683 (536)	32,548 (4441)	2665 (11)
Minocycline (MCL)	N.A.	4157 (905)	377 (36)
Tetracycline (TCL)	N.A.	N.A.	14 (6)
Vancomycin (VMC)	158 (25)	2905 (186)	169 (7)

*: Antimicrobial usage in Japan, **: Antimicrobial usage in the target area in this study, N.A.: Not available.

**Table 4:** Predicted concentrations of antimicrobials in the target STP influent.

Antimicrobials	Predicted Concentrations in the Targeted STP Influent (ng/L) (Mean (SD))		
	Sales	Shipping	NDB
Ampicillin (APL)	99 (17)	N.A.	78 (10)
Cefdinir (CDN)	37 (2.4)	87 (12)	77 (19)
Cefpodoxime proxetil (CPXP)	20 (5.8)	33 (12)	39 (3.4)
Ciprofloxacin (CFX)	N.A.	29 (5.1)	44 (4.2)
Clarithromycin (CTM)	117 (40)	724 (152)	790 (0.1)
Doxycycline (DCL)	N.A.	N.A.	48 (4.8)
Levofloxacin (LFX)	59 (19)	1134 (155)	1335 (4.9)
Minocycline (MCL)	N.A.	63 (14)	82 (7.9)
Tetracycline (TCL)	N.A.	N.A.	4.9 (1.8)
Vancomycin (VMC)	5.6 (0.9)	104 (6.6)	87 (4.0)

N.A.: Not available.
